# The Metabolic Response to Ozone

**DOI:** 10.3389/fimmu.2019.02890

**Published:** 2019-12-06

**Authors:** Stephanie A. Shore

**Affiliations:** Department of Environmental Health, Harvard T.H. Chan School of Public Health, Boston, MA, United States

**Keywords:** obesity, metabolome, microbiome, fatty acids, hyperglycemia

## Abstract

The respiratory effects of O_3_ are well established. High ambient O_3_ concentrations are associated with respiratory symptoms, declines in pulmonary function, asthma exacerbations, and even mortality. The metabolic effects of O_3_ are less well appreciated. Here we review data indicating that O_3_ exposure leads to glucose intolerance and hyperlipidemia, characteristics of the metabolic syndrome. We also review the role of stress hormones in these events. We describe how the metabolic effects of O_3_, including effects within the lungs, are exacerbated in the setting of the metabolic derangements of obesity and we discuss epidemiological data indicating an association between ambient O_3_ exposure and diabetes. We conclude by describing the role of the gut microbiome in the regulation of metabolism and by discussing data indicating a link between the gut microbiome and pulmonary responses to O_3_.

## Introduction

Ozone (O_3_) is an air pollutant produced by exposure of automobile exhaust to sunlight. The respiratory effects of O_3_ are well established. O_3_ causes peroxidation of lipids in the nasal and airway lining liquid and epithelial cell membranes, leading to epithelial cell damage and subsequent sterile inflammation (1, 2). The inflammatory response to ozone includes production of inflammatory cytokines and chemokines as well as activation of innate lymphoid cells type 2 and subsequent release of type 2 cytokines (3–5). The details of these events vary with the concentration of O_3_ and with the chronicity of exposure and the reader is referred to other reviews (1, 2) for an in depth description of these events. The net effect is that O_3_ causes respiratory symptoms including cough, shortness of breath, and wheezing, as well as declines in pulmonary function. O_3_ also increases the risk of pulmonary infections, of asthma exacerbations, and even of mortality, the latter mostly in patients with pre-existing cardiorespiratory conditions (6–10). What is less well-appreciated is that O_3_ also has pronounced metabolic effects. Here we review data indicating that O_3_ exposure impacts the function of the primary organs regulating metabolism leading to glucose intolerance and hyperlipidemia, characteristics of the metabolic syndrome. We describe how the effects of O_3_, including effects on the lungs, are exacerbated in the setting of the metabolic derangements of obesity. We conclude by describing the next frontier. The gut microbiome is a key player in the regulation of metabolism. There is increasing evidence of a role for the microbiome in pulmonary responses to O_3._ Whether the microbiome also contributes to the metabolic responses to O_3_ remains to be established.

## Acute Exposure to O_3_ Decreases the Metabolic Rate in Rodents

Almost four decades ago, Clemons and Garcia reported that acute O_3_ exposure reduces plasma concentrations of thyroid hormones (11). Consistent with the role of thyroid hormones in setting the metabolic rate, acute O_3_ exposure also reduces core body temperature, heart rate, activity level, food consumption, and minute ventilation (12–16). These changes are proportional to the O_3_ concentration administered and wane over time with repeated exposure. The reduction in minute ventilation that accompanies the reduction in metabolic rate would be expected to reduce the inhaled dose of O_3_ and has consequently been viewed as protective against the toxic effects of O_3_. Indeed, conditions that increase thyroid hormones, and thus increase the metabolic rate, including reductions in the ambient temperature and exogenous administration of thyroid hormones, and conditions that increase the metabolic rate, such as immaturity, increase the pulmonary inflammation and injury induced by acute O_3_ exposure (14, 15, 17).

The torpor-like state described above is similar to what is observed in rodents during acute fasting (18), and acute O_3_ exposure also has metabolic consequences similar to those observed during fasting: the adipose tissue initiates lipolysis mobilizing fatty acids that provide a source of energy, and the liver alters its handling of glucose. A metabolomic analysis of serum harvested from rats exposed to air or to O_3_ (1 ppm) indicates that short and long-chain free fatty acids (FFAs) are uniformly elevated after O_3_ exposure (19). Gene expression analysis on the livers from these rats indicated that O_3_ exposure alters many genes involved in fatty acid metabolism and insulin signaling. Last et al. (20) also reported changes in genes related to lipid and fatty acid metabolism and to carbohydrate metabolism in livers from air vs. O_3_ exposed mice. In addition, glucose tolerance tests performed on rats immediately after exposure to O_3_ indicate hyperglycemia and impaired glucose clearance (19, 21, 22). Similarly, serum 1,5-anhydroglucitol, which is inversely related to long-term glucose control, is decreased in O_3_-exposed rats (19). The effects of O_3_ are concentration dependent: little effect is observed at 0.25 ppm, glucose intolerance is observed after 0.5 ppm, and both fasting hyperglycemia and glucose intolerance are observed after 1 ppm exposure (23). The latter concentration is higher than would be experienced by humans even in the most polluted of cities, but there are differences in O_3_ dosimetry between rodents and humans (24). Importantly, glucose intolerance is also observed when rats are exposed to lower concentrations of O_3_ for more extended periods of time (25). Serum insulin is also elevated after O_3_ suggesting insulin resistance rather than impaired insulin release and experiments using euglycemic clamps verify insulin resistance (22). Acute O_3_ exposure also causes reduced insulin sensitivity in liver and skeletal muscle but not adipose tissue harvested from O_3_-exposed rats, as assessed by phosphorylation of AKT (21, 22).

There are sex differences in the metabolic response to acute O_3_ exposure. Gordon et al. (26) reported that male rats developed the same fasting hyperglycemia and glucose intolerance after acute O_3_ as described above, whereas females that were littermates of these males did not. In addition, although glucose tolerance tests performed after O_3_ exposure indicated some glucose intolerance in females, the effect was much smaller than was observed in males. Interestingly, markers of O_3_-induced pulmonary injury and inflammation were also lower in the female than male rats suggesting a link between the metabolic and inflammatory responses to O_3_.

## Evidence of Metabolic Effects of O_3_ in Humans

The metabolic effects of acute O_3_ exposure are not restricted to rodents. Miller et al. (27) performed a metabolomic analysis of serum collected 1 h after exposure of human subjects to filtered air or to O_3_ (0.3 ppm) with a 15-min on-off exercise cycle. The results indicated O_3_-induced increases in medium and long chain fatty acids and glycerol indicative of lipid mobilization from adipose tissue stores similar to what is observed in rats (19). Epidemiological studies also provide increasing evidence of an association between O_3_ exposure and diabetes. For example, in a large study from Italy that included 376,157 individuals, the authors noted a positive association between average annual ambient O_3_ concentrations and the risk of diabetes (28). A large (45,231 women) longitudinal analysis of African American women conducted over 16 years also indicated an association between ambient O_3_ concentrations and the risk of incident diabetes (29). The association remained unaltered even after controlling for particulate air pollution, which is also associated with diabetes (30). No association between O_3_ and hyperglycemia was observed in a study from the Framingham Heart cohort, even though associations were observed with PM_2.5_ and NO_2_, but the study was much smaller (5958 participants) and may not have been adequately powered to detect an association.

## Mechanistic Basis for the Metabolic Effects of O_3_

**Figure 1:**
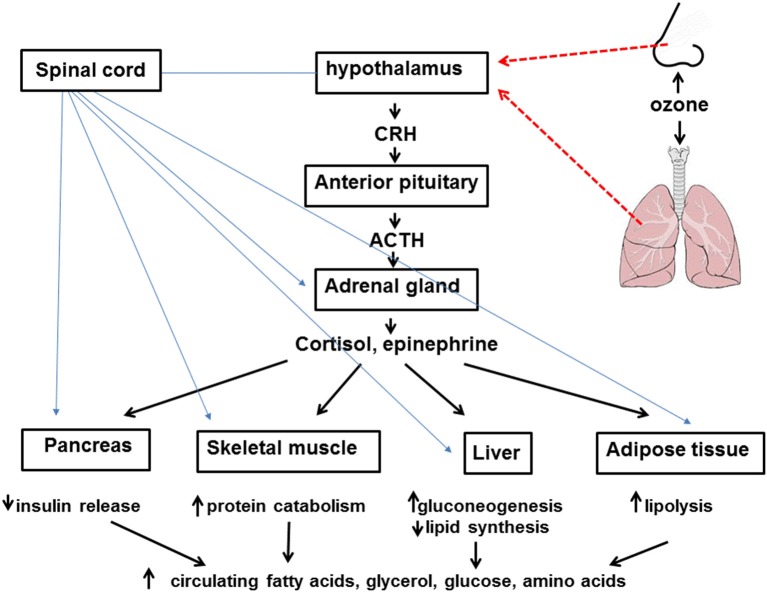
Inhaled ozone stimulates sensory afferents in the nose and lungs (hatched red lines) ultimately leading to activation of the hypothalamus and activation of the sympathetic nervous system as well as release of corticotropin releasing hormone (CRH). CRH leads to release of adrenocorticotropic hormone (ACTH) from the anterior pituitary which acts on the adrenal cortex to cause release of cortisol. Activation of the sympathetic nervous system (blue lines) leads to release of epinephrine from the adrenal medulla. Cortisol, epinephrine, and the sympathetic nervous system lead to attenuated insulin release from the pancreas, increased protein catabolism within skeletal muscle, reduced lipid synthesis and increased gluconeogenesis in the liver, and lipolysis within adipose tissue. The net effect of these events is increased circulating levels of fatty acids, glycerol, glucose, and amino acids.

Stress hormones likely account for the metabolic effects of O_3_ (Figure 1). In rodents, serum corticosterone levels increase immediately following acute O_3_ exposure (19, 31). A similar increase in cortisol is observed in human subjects after acute O_3_ exposure (27). Serum concentrations of epinephrine are also increased following O_3_ (19, 21, 31) and remain elevated even 18 h after cessation of exposure (21). These changes in stress hormones are thought to arise from O_3_-induced stimulation of sensory afferents within the lungs and nose (32). These afferents have been shown to terminate in stress responsive regions of the brain (33). Importantly, the hyperglycemia and impaired glucose clearance observed after acute O_3_ exposure are virtually abolished in rats in which either the adrenal medulla or the entire adrenal gland is surgically removed bilaterally (31). Acute O_3_-induced increases in serum lipids are also ablated by removal of either the adrenal medulla or the whole adrenal gland. The data are consistent with the known effects of epinephrine and cortisol in promoting gluconeogenesis, insulin resistance, and lipolysis in the liver and adipose tissue during fasting.

It is has been reported that adrenalectomy and drugs that block either beta adrenergic receptors or glucocorticoid signaling attenuate the pulmonary inflammation and injury that occur with O_3_ exposure (31, 34, 35). The genes whose expression in the lungs are impacted by O_3_ are similar to the genes whose expression changes with glucocorticoids or with agents that, like epinephrine, induce cAMP activation (34). Furthermore, adrenalectomy substantially reduces O_3_-induced changes in gene expression within the lungs (34). O_3_ exposure causes activation of NF-κB in the lungs and subsequent induction of a variety of inflammatory cytokines and chemokines that contribute to neutrophil recruitment (2, 36) and these events are inhibited by exogenous administration of dexamethasone (37, 38). Therefore, it is unlikely that the effects of adrenalectomy on O_3_-induced changes in gene expression (34) are the result of loss of the effects of stress hormones on activation of inflammatory genes by O_3_. Instead, the observed reductions in O_3_-induced neutrophil recruitment that occur after adrenalectomy or after inhibition of endogenous stress hormones (31, 34, 35) may be the result of inhibition of the effects of stress hormones on metabolic pathways. Inhibition of O_3_-induced NF-κB by corticosteroids occurs at higher concentrations of these steroids than are typically released endogenously following O_3_. In this context, it is interesting to note that fatty acids that are released in a stress-hormone dependent manner following O_3_ (31) should have the capacity activate neutrophils via the fatty acid receptor, FFAR1 (39).

Other events may also contribute to the metabolic effects of O_3_. O_3_ causes an inflammatory response in the lung characterized by release of acute phase cytokine and cytokines and increases in BAL neutrophils and macrophages (5). Lung specific overexpression of a constitutively active inhibitor of κB kinase (IKK2) not only causes a similar inflammatory response in the lungs, but also induces insulin resistance, perhaps by inducing systemic and adipose tissue inflammation (40), which are thought to mediate the insulin resistance associated with obesity (41). However, in mice, inhalation of another air pollutant, PM_2.5_, also causes inflammation with adipose tissue and liver, and leads to insulin resistance but the insulin resistance is not attenuated when the hepatic and adipose tissue inflammation are ameliorated by genetic deficiency in CCR2, the receptor for the macrophage chemotactic cytokines, CCL2 (42).

## Effects of O_3_ in Animals With Metabolic Syndrome

Given the effects of acute O_3_ exposure on lipid and carbohydrate metabolism, it is interesting to consider differences in the response to O_3_ under circumstances in which lipid and carbohydrate metabolism are already compromised: metabolic syndrome and obesity. In Goto-Kakizaki rats, a model of non-obese type 2 diabetes, exposure to approximately 0.4 ppm O_3_ for 4 h results in decreased LDL cholesterol and neither hyperglycemia nor glucose intolerance (43). Unfortunately, no normal rats were included in the study and the concentration used was lower than the 0.5–1.0 ppm concentrations that evoked hyperlipidemia, hyperglycemia, and glucose intolerance in normal rats (19, 21–23), so it is difficult to determine whether the differences are the result of the diabetic state or the nature of the exposure. In contrast, chronic O_3_ exposure does appear to affect glucose metabolism in insulin-resistant, diabetes-prone KK mice (44). When these mice are repeatedly exposed to O_3_ (0.5 ppm, 4 h a day for 13 days), the mice develop even greater insulin resistance. There is marked fasting hyperglycemia even in air-exposed KK mice and O_3_ does not cause any further increases in baseline glucose but does decrease fasting insulin, suggesting impaired insulin release. Injection of insulin gradually reduces blood glucose in air-exposed KK mice, but after O_3_ exposure, no such reduction is observed. Increased insulin resistance is also observed in rats and mice with diet-induced obesity as well as in normal weight mice after chronic pulmonary exposure to another air pollutant, PM_2.5_ (45–47). Adipose tissue inflammation and systemic inflammation are typically observed in obese mice, and repeated exposure to O_3_ exacerbates this inflammation (44): the number of activated macrophages within adipose tissue and the number of circulating inflammatory monocytes are both elevated in O_3_- vs. air-exposed KK mice. Expression of adipose tissue inflammatory genes linked to insulin resistance, including TNFα, is also elevated in O_3_ vs. air exposed KK mice. Pulmonary exposure to another type of air pollution, silicon dioxide nanoparticles, also augments mRNA expression of pro-inflammatory genes within adipose tissue (48). The effects of repeated O_3_ exposure on adipose tissue inflammation and insulin release but not insulin sensitivity were also observed in another model of obese type 2 diabetes, KKAy mice (49). The mechanistic basis for the effects of exposure to air pollution on adipose tissue gene expression, including inflammatory gene expression are not well-understood, but it is conceivable that changes in the gut microbiome may contribute (see below).

We have established that the pulmonary inflammation and injury induced by acute O_3_ exposure are also increased in obese mice. This effect of obesity was observed in *ob/ob* and *db/db* mice which are obese because of a genetically deficiency in leptin or the leptin receptor, in mice obese because of a genetic deficiency in carboxypeptidase E (Cpe), an enzyme involved in processing neuropeptides related to eating behavior, and in mice diet-induced obesity (5, 50–54). These mice are also diabetic to varying degrees. There are marked effects of obesity on the serum and urinary metabolomes of humans, rats, and mice including changes in carbohydrate, lipid, and branched chain amino acid (BCAA) metabolism (55–57). Lungs of naive obese mice also exhibit metabolic changes, including changes in lipid, phospholipid, and cholesterol metabolism (58). As described above, O_3_ has substantive metabolic effects that may be linked to effects of O_3_ on the lung. To determine whether O_3_ also affects metabolic processes within the lungs and whether these effects of O_3_ were modified by obesity, we performed a metabolomic analysis of lung tissue from *db/db* and wildtype (WT) female mice exposed acutely to air or O_3_ (54). Our results indicated substantial differences in the lung metabolomes of air-exposed *db/db* and WT mice including increases in lipids and lung carbohydrates. It is possible that increases in these substances in the lungs are due to corresponding increases in the blood (57) and subsequent diffusion into the lung extracellular fluid. Acute O_3_ exposure also affected the lung metabolome and there were differential effects of O_3_ in *db/db* and WT mice. For example, we observed effects of O_3_ on the substrates used for energy production in the lungs and these effects differed in *db/db* and WT mice. In WT mice, O_3_ exposure reduced BCAA metabolites consistent with increased reliance upon BCAA catabolism for energy, but no such effect was observed in *db/db* mice. Instead, in *db/db* mice, O_3_ resulted in decreased long chain acylcarnitines consistent with increased reliance upon β-oxidation for energy after O_3_ exposure. Changes in lung lipids are also observed in monkeys after chronic exposure to lower concentrations of O_3_ (59, 60).

As discussed above, O_3_-induced increases in stress hormones appear to mediate the hyperglycemia and hyperlipidemia that occur with acute O_3_ exposure. Corticosteroids also promote β-oxidation (61) and attenuate BCAA catabolism (62), similar to the effects of O_3_ in *db/db* mice. In our metabolomic analysis, lung corticosterone was greater in O_3_- than air-exposed mice, presumably as a result of increases in serum corticosterone, but the effect of O_3_ on corticosterone was only significant in *db/db* mice (54). Thus, greater O_3_-induced increases in corticosterone in *db/db* than WT mice might account for the different effects of O_3_ on lung β-oxidation and BCAA metabolism observed in *db/db* vs. WT mice.

## O_3_ and the Microbiome: the Next Frontier

Data from animal models indicate that the gut microbiome contributes to variety of metabolic conditions including insulin resistance and also affects metabolic processes within the liver (63–68). For example, treatment with oral antibiotics attenuates both the glucose intolerance and the adipose tissue inflammation observed in obese mice (65). Germ free mice consuming a Western style diet are protected against the development of obesity and have changes in skeletal muscle and liver that promote fatty acid metabolism (64). One way that gut microbiota regulate metabolism is via the production of metabolites that can impact their host. For example, gut microbiota modify bile acids which signal in the intestines and liver to regulate lipid metabolism (68). Hence, it is possible that the gut microbiome also contributes to the changes in metabolism as well as to the changes in hepatic gene transcription observed following acute O_3_ exposure.

Data from our lab also indicate a role for the microbiome in the metabolic changes observed in the lungs after O_3_ exposure. Among the lung metabolites identified in the metabolomic profiling experiment described above were several that require bacteria for their generation in mammals (54). Notably, each of these bacterial-mammalian co-metabolites was affected by obesity, by O_3_ exposure, or by the combination of obesity and O_3_ exposure. It is perhaps not surprising that obesity affects metabolites of bacterial origin. The community structure of the gut microbiome is altered by obesity both in rodents and in humans [see (69) for review] and there are differences in the metabolomic profile of tissues and blood harvested from germ free vs. conventionally housed mice and from antibiotic-treated vs. control mice (70–72). Thus, gut bacteria-derived metabolites can enter the blood and most are small enough to diffuse from the blood into the lungs. It is more surprising that O_3_ also affects these metabolites. One potential explanation is the effects of O_3_ on the liver (19, 20), since generation of many of the bacterial-mammalian co-metabolites identified in the lungs requires a metabolic step that occurs in the liver. However, it is also conceivable that O_3_ alters either the gut or the lung microbiome. O_3_ also affects the nose (3), and O_3_-induced changes to the nasal microbiome could also contribute to responses to O_3_ by altering metabolites that stimulate nasal afferents and contribute to activation of the HPA axis (Figure 1).

Our data indicate that bacteria also contribute to pulmonary responses to acute O_3_ exposure (73). O_3_-induced airway hyperresponsiveness and O_3_-induced neutrophil recruitment are reduced in male C57BL/6 mice treated with antibiotics, as well as in germ free mice. Since these changes are observed both with antibiotics that can cross the intestines and enter the blood and with antibiotics that cannot, the data suggest that the origin of the bacteria involved in these events is the gut and not the lungs. Gut bacteria generate short chain fatty acids (SCFAs) from dietary fiber and our data suggest a role for SCFAs in the effects of the microbiome on responses to O_3_. We observed reductions in serum SCFAs only in mice treated with those antibiotics that attenuated responses to O_3_. Furthermore, exogenous administration of SCFAs via the drinking water and diets high in fermentable fiber that increased serum SCFAs also augmented responses to O_3_ (73). Together, our data support a role for the gut microbiome in pulmonary responses to O_3_. Whether the gut microbiome also contributes to the metabolic changes observed after O_3_ exposure and whether O_3_ itself has the capacity to alter the gut microbiome remains to be established.

## Summary

In rodents, acute O_3_ exposure causes endocrine and metabolic changes similar to those observed during fasting: stress hormones are released and act on the liver, adipose tissue, and skeletal muscle countering the effects of insulin and promoting lipolysis, thus providing a ready source of energy. However, O_3_ also lowers the metabolic rate, reducing the need for energy. The net effects of these changes are hyperglycemia and hyperlipidemia, characteristics of the metabolic syndrome. Similar, albeit attenuated effects are observed in rodents after repeated exposures at lower concentrations of O_3_, an exposure paradigm that perhaps better reflects human exposures to ambient O_3_. Humans do not experience the torpor-like state that characterizes rodents exposed to O_3_, but hyperlipidemia is also observed after acute exposure of human subjects to O_3_ and there is an increasing body of epidemiological data indicating an association between O_3_ exposure and diabetes. Indeed, in certain types of obese diabetic rodents, O_3_ exacerbates their already compromised insulin sensitivity and also induces adipose tissue and systemic inflammation, other characteristics of the metabolic syndrome. O_3_ also differentially affects both energy metabolism and inflammation within the lungs of obese diabetic vs. normal lean mice. Better understanding of the mechanistic basis for the effects of O_3_ on the liver and adipose tissue is needed to protect populations already at risk of metabolic disease.

There is increasing evidence that the gut microbiome contributes to energy regulation. It remains to be established whether the gut microbiome also contributes to the derangements in energy regulation that occur after O_3_ exposure, but there is evidence of a link between the gut microbiome and pulmonary responses to O_3_. Better understanding of this link could result in strategies to prevent or mitigate the deleterious effects of O_3_ not only on the lungs, but also on metabolic health.

## Author Contributions

The author confirms being the sole contributor of this work and has approved it for publication.

### Conflict of Interest

The author declares that the research was conducted in the absence of any commercial or financial relationships that could be construed as a potential conflict of interest.

## References

[1] Bromberg PA. Mechanisms of the acute effects of inhaled ozone in humans. Biochim Biophys Acta. (2016) 1860:2771–81. 10.1016/j.bbagen.2016.07.015 27451958

[2] Mumby S Chung KF Adcock IM. Transcriptional effects of ozone and impact on airway inflammation. Front Immunol. (2019) 10:1610. 10.3389/fimmu.2019.01610 31354743 PMC6635463

[3] Kumagai K Lewandowski R Jackson-Humbles DN Li N Van Dyken SJ Wagner JG. Ozone-induced nasal type 2 immunity in mice is dependent on innate lymphoid cells. Am J Respir Cell Mol Biol. (2015) 54:782–91. 10.1165/rcmb.2015-0118OC 26559808

[4] Yang Q Ge MQ Kokalari B Redai IG Wang X Kemeny DM. Group 2 innate lymphoid cells mediate ozone-induced airway inflammation and hyperresponsiveness in mice. J Allergy Clin Immunol. (2015) 137:571–8. 10.1016/j.jaci.2015.06.037 26282284 PMC4747855

[5] Mathews JA Krishnamoorthy N Kasahara DI Cho Y Wurmbrand AP Ribeiro L. IL-33 drives augmented responses to ozone in obese mice. Environ Health Perspect. (2017) 125:246–53. 10.1289/EHP272 27472835 PMC5289908

[6] Gent JF Triche EW Holford TR Belanger K Bracken MB Beckett WS. Association of low-level ozone and fine particles with respiratory symptoms in children with asthma. JAMA. (2003) 290:1859–67. 10.1001/jama.290.14.1859 14532314

[7] Bell ML Dominici F Samet JM. A meta-analysis of time-series studies of ozone and mortality with comparison to the national morbidity, mortality, and air pollution study. Epidemiology. (2005) 16:436–45. 10.1097/01.ede.0000165817.40152.85 15951661 PMC3581312

[8] Ito K De Leon SF Lippmann M. Associations between ozone and daily mortality: analysis and meta-analysis. Epidemiology. (2005) 16:446–57. 10.1097/01.ede.0000165821.90114.7f 15951662

[9] Stanek LW Brown JS Stanek J Gift J Costa DL. Air pollution toxicology–a brief review of the role of the science in shaping the current understanding of air pollution health risks. Toxicol Sci. (2011) 120(Suppl 1):S8–27. 10.1093/toxsci/kfq367 21147959

[10] Turner MC Jerrett M Pope CA III Krewski D Gapstur SM Diver WR. Long-term ozone exposure and mortality in a large prospective study. Am J Respir Crit Care Med. (2016) 193:1134–42. 10.1164/rccm.201508-1633OC 26680605 PMC4872664

[11] Clemons GK Garcia JF. Changes in thyroid function after short-term ozone exposure in rats. J Environ Pathol Toxicol. (1980) 4:359–69. 7441119

[12] Umezu T Suzuki AK Miura T Koizumi A. Effects of ozone and nitrogen dioxide on drinking and eating behaviors in mice. Environ Res. (1993) 61:51–67. 10.1006/enrs.1993.1049 8472677

[13] Jimba M Skornik WA Killingsworth CR Long NC Brain JD Shore SA. Role of C fibers in physiological responses to ozone in rats. J Appl Physiol. (1995) 78:1757–63. 10.1152/jappl.1995.78.5.1757 7544341

[14] Watkinson WP Wiester MJ Highfill JW. Ozone toxicity in the rat. I Effect of changes in ambient temperature on extrapulmonary physiological parameters. J Appl Physiol. (1995) 78:1108–20. 10.1152/jappl.1995.78.3.1108 7775305

[15] Shore SA Abraham JH Schwartzman IN Murthy GG Laporte JD. Ventilatory responses to ozone are reduced in immature rats. J Appl Physiol. (2000) 88:2023–30. 10.1152/jappl.2000.88.6.2023 10846014

[16] Gordon CJ Johnstone AF Aydin C Phillips PM MacPhail RC Kodavanti UP. Episodic ozone exposure in adult and senescent Brown Norway rats: acute and delayed effect on heart rate, core temperature and motor activity. Inhal Toxicol. (2014) 26:380–90. 10.3109/08958378.2014.905659 24779854

[17] Huffman LJ Beighley CM Frazer DG McKinney WG Porter DW. Increased susceptibility of the lungs of hyperthyroid rats to oxidant injury: specificity of effects. Toxicology. (2006) 225:119–27. 10.1016/j.tox.2006.05.008 16797819

[18] Swoap SJ Weinshenker D. Norepinephrine controls both torpor initiation and emergence via distinct mechanisms in the mouse. PLoS ONE. (2008) 3:e4038. 10.1371/journal.pone.0004038 19107190 PMC2602851

[19] Miller DB Karoly ED Jones JC Ward WO Vallanat BD Andrews DL. Inhaled ozone (O3)-induces changes in serum metabolomic and liver transcriptomic profiles in rats. Toxicol Appl Pharmacol. (2015) 286:65–79. 10.1016/j.taap.2015.03.025 25838073 PMC4765366

[20] Last JA Gohil K Mathrani VC Kenyon NJ. Systemic responses to inhaled ozone in mice: cachexia and down-regulation of liver xenobiotic metabolizing genes. Toxicol Appl Pharmacol. (2005) 208:117–26. 10.1016/j.taap.2005.02.001 16183385

[21] Bass V Gordon CJ Jarema KA MacPhail RC Cascio WE Phillips PM. Ozone induces glucose intolerance and systemic metabolic effects in young and aged Brown Norway rats. Toxicol Appl Pharmacol. (2013) 273:551–60. 10.1016/j.taap.2013.09.029 24103449 PMC4343260

[22] Vella RE Pillon NJ Zarrouki B Croze ML Koppe L Guichardant M. Ozone exposure triggers insulin resistance through muscle c-Jun N-terminal kinase activation. Diabetes. (2015) 64:1011–24. 10.2337/db13-1181 25277399

[23] Gordon CJ Phillips PM Ledbetter A Snow SJ Schladweiler MC Johnstone AF. Active vs. sedentary lifestyle from weaning to adulthood and susceptibility to ozone in rats. Am J Physiol Lung Cell Mol Physiol. (2017) 312:L100–9. 10.1152/ajplung.00415.2016 27836902

[24] Hatch GE Slade R Harris LP McDonnell WF Devlin RB Koren HS. Ozone dose and effect in humans and rats. A comparison using oxygen-18 labeling and bronchoalveolar lavage. Am J Respir Crit Care Med. (1994) 150:676–83. 10.1164/ajrccm.150.3.8087337 8087337

[25] Miller DB Snow SJ Henriquez A Schladweiler MC Ledbetter AD Richards JE. Systemic metabolic derangement, pulmonary effects, and insulin insufficiency following subchronic ozone exposure in rats. Toxicol Appl Pharmacol. (2016) 306:47–57. 10.1016/j.taap.2016.06.027 27368153 PMC5336346

[26] Gordon CJ Phillips PM Johnstone AFM Schmid J Schladweiler MC Ledbetter A. Effects of maternal high-fat diet and sedentary lifestyle on susceptibility of adult offspring to ozone exposure in rats. Inhal Toxicol. (2017) 29:239–54. 10.1080/08958378.2017.1342719 28819990

[27] Miller DB Ghio AJ Karoly ED Bell LN Snow SJ Madden MC. Ozone exposure increases circulating stress hormones and lipid metabolites in humans. Am J Respir Crit Care Med. (2016) 193:1382–91. 10.1164/rccm.201508-1599OC 26745856 PMC5440058

[28] Orioli R Cremona G Ciancarella L Solimini AG. Association between PM10, PM2.5, NO2, O3 and self-reported diabetes in Italy: a cross-sectional, ecological study. PLoS ONE. (2018) 13:e0191112. 10.1371/journal.pone.0191112 29342195 PMC5771616

[29] Jerrett M Brook R White LF Burnett RT Yu J Su J. Ambient ozone and incident diabetes: a prospective analysis in a large cohort of African American women. Environ Int. (2017) 102:42–7. 10.1016/j.envint.2016.12.011 28153529 PMC5542012

[30] Eze IC Hemkens LG Bucher HC Hoffmann B Schindler C Kunzli N. Association between ambient air pollution and diabetes mellitus in Europe and North America: systematic review and meta-analysis. Environ Health Perspect. (2015) 123:381–9. 10.1289/ehp.1307823 25625876 PMC4421762

[31] Miller DB Snow SJ Schladweiler MC Richards JE Ghio AJ Ledbetter AD. Acute ozone-induced pulmonary and systemic metabolic effects are diminished in adrenalectomized rats. Toxicol Sci. (2016) 150:312–22. 10.1093/toxsci/kfv331 26732886 PMC4881831

[32] Henriquez AR House JS Snow SJ Miller CN Schladweiler MC Fisher A Ozone-induced dysregulation of neuroendocrine axes requires adrenal-derived stress hormones. Toxicol Sci. (2019) 172:38–50. 10.1093/toxsci/kfz182 PMC9344225 31397875

[33] Gackiere F Saliba L Baude A Bosler O Strube C. Ozone inhalation activates stress-responsive regions of the CNS. J Neurochem. (2011) 117:961–72. 10.1111/j.1471-4159.2011.07267.x 21466555

[34] Henriquez A House J Miller DB Snow SJ Fisher A Ren H. Adrenal-derived stress hormones modulate ozone-induced lung injury and inflammation. Toxicol Appl Pharmacol. (2017) 329:249–58. 10.1016/j.taap.2017.06.009 28623178 PMC6757346

[35] Henriquez AR Snow SJ Schladweiler MC Miller CN Dye JA Ledbetter AD. Adrenergic and glucocorticoid receptor antagonists reduce ozone-induced lung injury and inflammation. Toxicol Appl Pharmacol. (2018) 339:161–71. 10.1016/j.taap.2017.12.006 29247675 PMC7110430

[36] Devlin RB McDonnell WF Mann R Becker S House DE Schreinemachers D. Exposure of humans to ambient levels of ozone for 6.6 hours causes cellular and biochemical changes in the lung. Am J Respir Cell Mol Biol. (1991) 4:72–81. 10.1165/ajrcmb/4.1.72 1846079

[37] Haddad EB Salmon M Koto H Barnes PJ Adcock I Chung KF. Ozone induction of cytokine-induced neutrophil chemoattractant (CINC) and nuclear factor-kappa b in rat lung: inhibition by corticosteroids. FEBS Lett. (1996) 379:265–8. 10.1016/0014-5793(95)01524-8 8603703

[38] Salmon M Koto H Lynch OT Haddad EB Lamb NJ Quinlan GJ. Proliferation of airway epithelium after ozone exposure: effect of apocynin and dexamethasone. Am J Respir Crit Care Med. (1998) 157(3 Pt 1):970–7. 10.1164/ajrccm.157.3.9704067 9517619

[39] Manosalva C Mena J Velasquez Z Colenso CK Brauchi S Burgos RA. Cloning, identification and functional characterization of bovine free fatty acid receptor-1 (FFAR1/GPR40) in neutrophils. PLoS ONE. (2015) 10:e0119715. 10.1371/journal.pone.0119715 25790461 PMC4366208

[40] Chen M Zhou H Xu Y Qiu L Hu Z Qin X From the cover: lung-specific overexpression of constitutively active IKK2 induces pulmonary and systemic inflammations but not hypothalamic inflammation and glucose intolerance. Toxicol Sci. (2017) 160:4–14. 10.1093/toxsci/kfx154 29036520 PMC5837620

[41] Arkan MC Hevener AL Greten FR Maeda S Li ZW Long JM. IKK-beta links inflammation to obesity-induced insulin resistance. Nat Med. (2005) 11:191–8. 10.1038/nm1185 15685170

[42] Liu C Xu X Bai Y Zhong J Wang A Sun L. Particulate Air pollution mediated effects on insulin resistance in mice are independent of CCR2. Part Fibre Toxicol. (2017) 14:6. 10.1186/s12989-017-0187-3 28253935 PMC5335830

[43] McGee Hargrove M Snow SJ Luebke RW Wood CE Krug JD Krantz QT. Effects of simulated smog atmospheres in rodent models of metabolic and immunologic dysfunction. Environ Sci Technol. (2018) 52:3062–70. 10.1021/acs.est.7b06534 29384667 PMC6233996

[44] Zhong J Allen K Rao X Ying Z Braunstein Z Kankanala SR. Repeated ozone exposure exacerbates insulin resistance and activates innate immune response in genetically susceptible mice. Inhal Toxicol. (2016) 28:383–92. 10.1080/08958378.2016.1179373 27240593 PMC4911226

[45] Sun Q Yue P Deiuliis JA Lumeng CN Kampfrath T Mikolaj MB. Ambient air pollution exaggerates adipose inflammation and insulin resistance in a mouse model of diet-induced obesity. Circulation. (2009) 119:538–46. 10.1161/CIRCULATIONAHA.108.799015 19153269 PMC3845676

[46] Xu X Liu C Xu Z Tzan K Zhong M Wang A. Long-term exposure to ambient fine particulate pollution induces insulin resistance and mitochondrial alteration in adipose tissue. Toxicol Sci. (2011) 124:88–98. 10.1093/toxsci/kfr211 21873646 PMC3196653

[47] Yan YH Chou CC Lee CT Liu JY Cheng TJ. Enhanced insulin resistance in diet-induced obese rats exposed to fine particles by instillation. Inhal Toxicol. (2011) 23:507–19. 10.3109/08958378.2011.587472 21736501

[48] Zhang Y Lin Y Li X Zhang L Pan W Zhu H. Silica dioxide nanoparticles combined with cold exposure induce stronger systemic inflammatory response. Environ Sci Pollut Res Int. (2017) 24:291–8. 10.1007/s11356-016-7649-2 27714660

[49] Ying Z Allen K Zhong J Chen M Williams KM Wagner JG Subacute inhalation exposure to ozone induces systemic inflammation but not insulin resistance in a diabetic mouse model. Inhal Toxicol. (2016) 28:155–63. 10.3109/08958378.2016.1146808 26986950 PMC4836866

[50] Shore SA Rivera-Sanchez YM Schwartzman IN Johnston RA. Responses to ozone are increased in obese mice. J Appl Physiol. (2003) 95:938–45. 10.1152/japplphysiol.00336.2003 12794034

[51] Johnston RA Theman TA Shore SA. Augmented responses to ozone in obese carboxypeptidase E-deficient mice. Am J Physiol Regul Integr Comp Physiol. (2006) 290:R126–33. 10.1152/ajpregu.00306.2005 16002559

[52] Johnston RA Theman TA Lu FL Terry RD Williams ES Shore SA. Diet-induced obesity causes innate airway hyperresponsiveness to methacholine and enhances ozone-induced pulmonary inflammation. J Appl Physiol. (2008) 104:1727–35. 10.1152/japplphysiol.00075.2008 18323466

[53] Williams AS Mathews JA Kasahara DI Chen L Wurmbrand AP Si H. Augmented pulmonary responses to acute ozone exposure in obese mice: roles of TNFR2 and IL-13. Environ Health Perspect. (2013) 121:551–7. 10.1289/ehp.1205880 23434795 PMC3673194

[54] Mathews JA Kasahara DI Cho Y Bell LN Gunst PR Karoly ED. Effect of acute ozone exposure on the lung metabolomes of obese and lean mice. PLoS ONE. (2017) 12:e0181017. 10.1371/journal.pone.0181017 28704544 PMC5509247

[55] Newgard CB An J Bain JR Muehlbauer MJ Stevens RD Lien LF. A branched-chain amino acid-related metabolic signature that differentiates obese and lean humans and contributes to insulin resistance. Cell Metab. (2009) 9:311–26. 10.1016/j.cmet.2009.02.002 19356713 PMC3640280

[56] Won EY Yoon MK Kim SW Jung Y Bae HW Lee D. Gender-specific metabolomic profiling of obesity in leptin-deficient ob/ob mice by 1H NMR spectroscopy. PLoS ONE. (2013) 8:e75998. 10.1371/journal.pone.0075998 24098417 PMC3789719

[57] Giesbertz P Padberg I Rein D Ecker J Hofle AS Spanier B. Metabolite profiling in plasma and tissues of ob/ob and db/db mice identifies novel markers of obesity and type 2 diabetes. Diabetologia. (2015) 58:2133–43. 10.1007/s00125-015-3656-y 26058503

[58] Milner JJ Rebeles J Dhungana S Stewart DA Sumner SC Meyers MH. Obesity increases mortality and modulates the lung metabolome during pandemic H1N1 influenza virus infection in mice. J Immunol. (2015) 194:4846–59. 10.4049/jimmunol.1402295 25862817 PMC4417391

[59] Rao GA Larkin EC Harkema JR Dungworth DL. Changes in lipids of lung lavage in monkeys after chronic exposure to ambient levels of ozone. Toxicol Lett. (1985) 29:207–14. 10.1016/0378-4274(85)90043-8 4089887

[60] Rao GA Larkin EC Harkema JR Dungworth DL. Changes in the levels of polyunsaturated fatty acids in the lung and lecithin cholesterol acyl transferase activity in plasma of monkeys exposed to ambient levels of ozone. Toxicol Lett. (1985) 24:125–9. 10.1016/0378-4274(85)90047-5 3983964

[61] Morgan SA Gathercole LL Simonet C Hassan-Smith ZK Bujalska I Guest P. Regulation of lipid metabolism by glucocorticoids and 11beta-HSD1 in skeletal muscle. Endocrinology. (2013) 154:2374–84. 10.1210/en.2012-2214 23633532

[62] Nawabi MD Block KP Chakrabarti MC Buse MG. Administration of endotoxin, tumor necrosis factor, or interleukin 1 to rats activates skeletal muscle branched-chain alpha-keto acid dehydrogenase. J Clin Invest. (1990) 85:256–63. 10.1172/JCI114421 2404025 PMC296413

[63] Dumas ME Barton RH Toye A Cloarec O Blancher C Rothwell A. Metabolic profiling reveals a contribution of gut microbiota to fatty liver phenotype in insulin-resistant mice. Proc Natl Acad Sci USA. (2006) 103:12511–6. 10.1073/pnas.0601056103 16895997 PMC1567909

[64] Backhed F Manchester JK Semenkovich CF Gordon JI. Mechanisms underlying the resistance to diet-induced obesity in germ-free mice. Proc Natl Acad Sci USA. (2007) 104:979–84. 10.1073/pnas.0605374104 17210919 PMC1764762

[65] Cani PD Delzenne NM Amar J Burcelin R. Role of gut microflora in the development of obesity and insulin resistance following high-fat diet feeding. Pathol Biol. (2008) 56:305–9. 10.1016/j.patbio.2007.09.008 18178333

[66] Compare D Coccoli P Rocco A Nardone OM De Maria S Carteni M. Gut–liver axis: the impact of gut microbiota on non alcoholic fatty liver disease. Nutr Metab Cardiovasc Dis. (2012) 22:471–6. 10.1016/j.numecd.2012.02.007 22546554

[67] Kimura I Ozawa K Inoue D Imamura T Kimura K Maeda T. The gut microbiota suppresses insulin-mediated fat accumulation via the short-chain fatty acid receptor GPR43. Nat Commun. (2013) 4:1829. 10.1038/ncomms2852 23652017 PMC3674247

[68] Wahlstrom A Sayin SI Marschall HU Backhed F. Intestinal crosstalk between bile acids and microbiota and its impact on host metabolism. Cell Metab. (2016) 24:41–50. 10.1016/j.cmet.2016.05.005 27320064

[69] Shore SA Cho Y. Obesity and asthma: microbiome-metabolome interactions. Am J Respir Cell Mol Biol. (2016) 54: 609–17. 10.1165/rcmb.2016-0052PS 26949916 PMC4942201

[70] Wikoff WR Anfora AT Liu J Schultz PG Lesley SA Peters EC. Metabolomics analysis reveals large effects of gut microflora on mammalian blood metabolites. Proc Natl Acad Sci USA. (2009) 106:3698–703. 10.1073/pnas.0812874106 19234110 PMC2656143

[71] Holmes E Li JV Marchesi JR Nicholson JK. Gut microbiota composition and activity in relation to host metabolic phenotype and disease risk. Cell Metab. (2012) 16:559–64. 10.1016/j.cmet.2012.10.007 23140640

[72] El Aidy S Derrien M Merrifield CA Levenez F Dore J Boekschoten MV. Gut bacteria-host metabolic interplay during conventionalisation of the mouse germfree colon. ISME J. (2013) 7:743–55. 10.1038/ismej.2012.142 23178667 PMC3603396

[73] Cho Y Abu-Ali G Tashiro H Kasahara DI Brown TA Brand JD. The microbiome regulates pulmonary responses to ozone in mice. Am J Respir Cell Mol Biol. (2018) 59:346–54. 10.1165/rcmb.2017-0404OC 29529379 PMC6189641

